# Introducing a foundational sequence transformer for range adaptive nucleotide decoding (STRAND)

**DOI:** 10.1093/bib/bbaf618

**Published:** 2025-11-24

**Authors:** Shant Ayanian, Collin Osborne, Clark Xu, Carl Molnar, Pravat Das, Xoab Perez, Natalia Vassilieva, Vinay Pondenkandath, Bhargav Kanakiya, Ganesh Venkatesh, May Levin, Matt Redlon, Marc Blasi, Vijay H Shah, Matthew Callstrom, Konstantinos N Lazaridis, Panos Korfiatis, Alexander Ryu, Elena Myasoedova

**Affiliations:** Mayo Clinic, 200 1st St SW, Rochester 55905, MN, United States; Mayo Clinic, 200 1st St SW, Rochester 55905, MN, United States; Mayo Clinic, 200 1st St SW, Rochester 55905, MN, United States; Mayo Clinic, 200 1st St SW, Rochester 55905, MN, United States; Mayo Clinic, 200 1st St SW, Rochester 55905, MN, United States; Mayo Clinic, 200 1st St SW, Rochester 55905, MN, United States; Cerebras Systems, Inc., 1237 E. Arques Ave, Sunnyvale 94085, CA, United States; Cerebras Systems, Inc., 1237 E. Arques Ave, Sunnyvale 94085, CA, United States; Cerebras Systems, Inc., 1237 E. Arques Ave, Sunnyvale 94085, CA, United States; Cerebras Systems, Inc., 1237 E. Arques Ave, Sunnyvale 94085, CA, United States; Cerebras Systems, Inc., 1237 E. Arques Ave, Sunnyvale 94085, CA, United States; Mayo Clinic, 200 1st St SW, Rochester 55905, MN, United States; Mayo Clinic, 200 1st St SW, Rochester 55905, MN, United States; Mayo Clinic, 200 1st St SW, Rochester 55905, MN, United States; Mayo Clinic, 200 1st St SW, Rochester 55905, MN, United States; Mayo Clinic, 200 1st St SW, Rochester 55905, MN, United States; Mayo Clinic, 200 1st St SW, Rochester 55905, MN, United States; Mayo Clinic, 200 1st St SW, Rochester 55905, MN, United States; Mayo Clinic, 200 1st St SW, Rochester 55905, MN, United States

**Keywords:** exome, transformer architecture, machine learning, benchmarking, rheumatoid arthritis

## Abstract

The advent of high-throughput sequencing has led to an exponential increase in genomic data, highlighting the need for efficient and accurate models to analyze and interpret this information. In this study, we introduce a novel, exomic foundational model that leverages a combination of the human reference genome and multispecies data to improve variant detection and interpretation. Our model utilizes a short-range transformer architecture and is trained on a large dataset of human exomic sequences derived from the Tapestry study. Through a series of ablation studies and scaling experiments, we demonstrate the effectiveness of our model in predicting next token accuracy and identifying clinically pathogenic variants. We also show that our model outperforms existing models in a range of downstream tasks, including variant effect prediction and disease state identification. In fact, our largest sequence transformer for range adaptive nucleotide decoding variant (1B parameters) surpassed previous benchmarks, demonstrating a mean accuracy of 0.880 [an 8.2% improvement over the original nucleotide transformer (NT) and a 7% improvement over NT-v2]. Furthermore, we construct a unique exomic ClinVar dataset to evaluate the model’s performance on pathogenicity and disease states. Our results highlight the potential of this model to improve our understanding of the human exome and its role in disease. The model and its applications have significant implications for genomics-based diagnosis and personalized medicine, including tailored therapeutic development.

## Introduction

Next-generation sequencing (NGS) strategies have revolutionized the field of genomics by significantly increasing the speed and reducing the cost of DNA sequencing. This has enabled large-scale genomic projects, such as the 1000 Genomes Project, to be completed more efficiently, providing deeper insights into genetic variation and disease mechanisms [1]. In addition, NGS has facilitated the development of personalized medicine by allowing the identification of pathogenic genetic variants associated with diseases of individual patients, leading to more targeted and effective treatments [2]. NGS has led to an unprecedented accumulation of genomic data that requires the development of advanced bioinformatics tools and computational infrastructure to efficiently store, manage, and analyze information [3, 4].

New computational methodologies such as deep learning networks and neural networks have been implemented over the past 10 years to enable the use of these accumulated data in novel ways, making knowledge more accessible and faster to use. These models excel in predicting the impact of genetic variations on gene regulatory mechanisms, such as DNA accessibility and splicing [5–9]. The shift from traditional neural networks to transformers in genomics has been driven by the unique advantages that transformers can offer. Research on the best architectures of large language models (LLMs) for different fields has accelerated since 2020. In fact, researchers have been able to train new LLMs with domain-specific applications in fields such as clinical medicine, radiology, and oncology [10, 11]. The newer LLMs released in 2024 have demonstrated excellent adaptability within medical applications, but research is still underway to understand the need and benefit of domain-adapted LLMs [12].

Over the last few years, diverse approaches have highlighted ongoing efforts to tailor transformer architectures to the unique challenges of genomic data analysis. During the last three years, some powerful genomic LLMs have been released that test this approach with significant promise [13–19]. The emerging clinical application potential of such models in patients with chronic diseases with high clinical and genetic heterogeneity has not yet been tested.

To address these gaps in the knowledge of clinically applicable artificial intelligence (AI)-based genetic solutions, our objective was to (i) develop and validate a novel exomic transformer to process and interpret upstream exomic information (aligned reads), and (ii) apply the nucleotide transformer (NT) to identify sequences associated with two autoimmune diseases, rheumatoid arthritis (RA) and inflammatory bowel disease (IBD), as clinical use cases. This new transformer, using whole exomes rather than single nucleotide polymorphisms and specific genes, is poised to create a novel way to interpret whole exomic information and offers a completely new way of addressing the problem of identification of patients with specific phenotypes, prediction of treatment response, and disease outcomes.

RA is a genetically complex polygenic chronic inflammatory autoimmune disease with a prevalence of 0.5–1% and an incidence of 20–50 per 100 000 annually [20, 21]. Untreated and/or uncontrolled inflammation in RA is associated with high rates of disability, an increased burden of comorbidity, and mortality [22–24]. Despite the major advancements in immunosuppressive treatments and advent of targeted therapies, only about 15% of patients achieve early sustained remission while most patients continue to experience moderate to high disease activity and disease flares [25–27]. Phenotypic heterogeneity, high genetic complexity, and incomplete understanding of RA pathogenesis contribute to the lack of robust predictive approaches for efficient and effective tailoring of RA treatments to an individual patient. Although several clinical and genomic markers of patient response to various RA treatments have been proposed, clinically applicable solutions are few [28].

IBD is a group of chronic, relapsing inflammatory disorders of the gastrointestinal tract, primarily comprising Crohn’s disease and ulcerative colitis. Globally, the prevalence of IBD has risen substantially over recent decades, now affecting an estimated 0.6%–0.8% of people in North America and Europe, with 3.1 million people are currently living with IBD [29, 30]. Accurate diagnosis of IBD remains challenging due to symptom overlap with other gastrointestinal conditions and the variable presentation of disease, often leading to delayed identification. Furthermore, treatment is complicated by disease heterogeneity, suboptimal response rates, and frequent therapeutic failures, even with advanced biologic and small-molecule therapies [31]. While more than 240 genetic susceptibility loci for IBD have been identified, translation of this genetic knowledge into reliable, personalized diagnostic or therapeutic strategies is still lacking, limiting the immediate clinical utility of genetic information in routine practice [32].

## Results

We present the results of the evaluation of architectural innovations and performance in established genomic benchmark datasets and novel clinically relevant variant interpretation tasks with STRAND (Sequence Transformer for Range Adaptive Nucleotide Decoding). Detailed ablation studies supporting these design choices are presented in Section 0.0.6 and Supplementary material.

### STRAND design and performance analysis

STRAND is a transformer-based foundation model designed to process and understand aligned upstream genomic sequencing data directly, without requiring traditional pre-processing steps [8]. The development of a model capable of learning directly from upstream genomic sequences represents a significant departure from conventional genomic analysis pipelines and presented several technical challenges that influenced our architectural decisions and training methodology. We describe these challenges and summarize our solutions below, followed by a comprehensive performance analysis.

- **Information organization:** Transformers excel at learning from long sequences of input data. However, aligned and unprocessed genome sequencing data comes in the form of short 151-base-pair reads that cannot be trivially stitched together into a continuous sequence. This fragmented nature of the input data requires the development of data packing and training recipes that enable the model to effectively understand genetic information across discontinuous segments while maintaining the ability to capture long-range dependencies.
- **Data redundancy:** Sequencing reads contain significant redundancy, with a coverage depth varying from 1 to 180 reads for each genomic position. Within each read of approximately 151 base-pairs, typically only 1–2 base pairs differ from the human reference genome (HRG) at any given position. This redundancy is a natural consequence of the shotgun sequencing process and necessitates careful data filtering and processing strategies to maintain computational efficiency without losing important variant information.
- **Signal-to-noise ratio:** When training a model to understand genetic variants, the actual variants represent a very small fraction of the total base pairs in the data. This creates a significant imbalance where the model could trivially achieve high accuracy by memorizing the more frequent HRG sequence while ignoring the rare but important nucleotide variants. To address this, specialized training strategies ensure that the model pays appropriate attention to variant positions despite their low frequency in the training data.

These challenges significantly influenced the architectural decisions and training methodology. Below, we describe the final architecture of STRAND and present comprehensive experimental results demonstrating how the design choices address each challenge. In particular, we discuss the specific design choices made to address the challenges outlined above in the first three subsections (see Sequence packing, Data filtration, Importance weighting) and finish by discussing the model architecture to build a scalable model design (see Model attention design for scalability).

#### Sequence packing

Raw sequencing data consist of short, 151-base-pair reads, unlike a contiguous reference genome. These reads are associated with positional metadata, which were leveraged to pack them into a fixed-size context window. Several packing strategies were explored:

- **Consecutive packing:** Reads from the same chromosome were packed into a single context in ascending order of their genomic position.
- **Random packing:** Reads from the same chromosome were randomly packed into a single context.
- **Overlapping packing:** Adjacent reads were merged into the longest possible contiguous sequences and packed into a single context.
- **Spaced packing:** Adjacent reads without overlap were packed into a single context ensuring a more uniform distribution of genomic positions within the context.

The performance of these packing strategies is summarized in Table 1. We performed an all-to-all comparison, training models on data prepared with each packing strategy and evaluating them on datasets prepared with each strategy, as well as the HRG dataset (GRCh38.p14, Human Genome Overview – Genome Reference Consortium). The evaluation criteria in this case was the model’s accuracy to predict the next base pair based on a given sequence. Surprisingly, the model trained with consecutive packing yielded the best performance across all packing schemes, outperforming models trained with the other packing strategies. This result was unexpected, as our original hypothesis was that the overlapping or spaced packing scheme would perform better because it incorporates domain-specific knowledge and packs more unique information within a context.

**Table 1:** Next-token prediction accuracy (%) for different sequence packing strategies

**Test set**	**HRG**	**HRG + Packing strategy**			
	**Only**	**Random**	**Consecutive**	**Spaced**	**Overlap**
HRG	58.47	56.48	58.67	57.66	57.38
Random	40.73	42.11	42.95	41.76	41.48
Consecutive	83.97	48.36	87.28	83.94	84.21
Cons. spaced	58.32	49.39	51.60	61.01	50.44
Cons. overlap	50.63	48.08	61.40	50.86	58.43

This superior performance of consecutive packing suggests that maintaining local genomic context is more important for model learning than maximizing the diversity of positions within each training window. This finding informed our final model architecture and training protocol.

#### Data filtration

To mitigate data redundancy and enhance the signal-to-noise ratio, we implemented a data filtration strategy designed to remove low-quality reads, thereby retaining only the most informative data. This approach enabled an 8-fold reduction in the number of reads used for training, while maintaining comparable next-token prediction accuracy. As shown in Table 2, the $8\times $ filtered model achieves performance on par with the unfiltered model, despite utilizing significantly less data. These results highlight the efficacy of our data filtration strategy in reducing computational demands without compromising model accuracy.

**Table 2:** Next token prediction accuracy (%) across different datasets and experimental conditions

Dataset	HRG	Tapestry + HRG					
		1 subj.	25 subjects				100 subjects
			Unfilt.	8x filt.	8x filt. +HRG	8x filt. + HRG + dyn.	8x filt. + HRG + dyn.
HRG	58.29	58.15	58.17	56.04	58.10	58.87	58.89
Tapestry 25	42.05	42.88	42.75	42.26	43.09	43.17	43.31
Tapestry 100	42.02	42.89	42.64	42.14	42.98	43.11	43.26

Tapestry 25: refers to a random mix of 25 participants from Tapestry. Tapestry 100 refers to a random mix of 100 participants from Tapestry. dyn refers to dynamic loss weighting.

#### Importance weighting

The inherent low signal-to-noise ratio (see STRAND design and performance analysis) within the sequencing data posed a challenge to effective model training. The default training strategy tends to assign higher importance to more frequently occurring patterns, but this assumption does not hold true for genomic sequences where infrequently occurring mutations likely carry crucial semantic meaning. To address this, we implemented a dynamic importance re-weighting strategy where we assign an “importance” weight to each base pair, which is described in further detail in section Methods. In terms of implementation, we apply this “importance” weighting of each base pair by adjusting the loss function of the STRAND model training and hence term this technique as “dynamic loss weighting”.

As demonstrated in Table 2, increased filtration – critical for removing redundancy and improving training efficiency – results in degraded performance on the reference genome, going from 58.29 when training exclusively on the reference genome to 56.04 when using 8x filtration with 25 subject exomes. This degradation in performance is partly recovered by incorporating additional versions of the complete reference genome to increase the relative proportion of the reference genome relative to exome data for 25 subjects. However, this approach is limited, as scaling to an increasing number of subjects would require significantly more tokens of the reference genome, which can lead to suboptimal learning behavior. Dynamic loss weighting of each token based on its relative presence in the training data allows for normalization and significantly greater control over learning dynamics. Notably, the accuracy on the HRG dataset is improved, with the dynamically weighted model achieving a score of 0.5889, surpassing even the performance of the model trained on the unfiltered HRG dataset (0.5829). This highlights the efficacy of our dynamic loss weighting approach in counteracting the effects of data filtration and improving overall model performance.

#### Model attention design for scalability

Given the significant increase in genomic data available for training, the design choice of BERT vs GPT for genomics data requires additional thought. In particular, in a comparative experiment using both GPT-style and BERT-style attention mechanisms as shown in Table 3, the GPT-style attention model achieved a higher token prediction accuracy compared to the BERT-style attention model, while maintaining comparable computational cost. Consequently, we selected GPT-style attention for the STRAND architecture.

**Table 3:** Comparison of GPT and BERT style attention mechanisms

**Attention style**	**Accuracy (%)**
GPT (causal attention)	58.75
BERT (MLM with 15% masking)	43.41

**Data scaling:** We evaluate our model scalability both with respect to data quantity as well as data diversity. We do so by scaling the input data from just being HRG to the more complex Tapestry data, where we increase the number of subjects from 1 to 100. As we show in Table 2, increasing the number of training subjects generally improves next token prediction accuracy across datasets.

Finally, to assess the impact of broader genomic context, we incorporated multispecies genomes into the training data. Table 4 shows that including multispecies genomes positively impacted model performance on the HRG dataset. This highlights the model’s capacity to generalize and learn from the expanded genomic context offered by multispecies data. This generalization ability not only enhances the model’s robustness but also suggests its potential for wider applicability across different species and genomic datasets.

**Table 4:** Impact of multispecies genome incorporation on model performance, next token prediction accuracy (%)

**Dataset**	**HRG only**	**HRG + MS**	
		50:50, 15% of MS	14:86, 100% of MS
HRG	58.29	58.83	58.87

MS: multispecies. 50:50 refers to 50% of total input data as HRG and 50% MS, while 15% refers to the percentage of the total MS data available.

### Model performance

To comprehensively evaluate the capabilities of STRAND, we assessed its performance across two key dimensions:

- **Genomic structural understanding,** specifically targeted the structural intricacies of genomes through its testing against the NT benchmarks [14].
- **Exome-specific understanding.** With the ultimate objective of leveraging STRAND for improved human health predictions, we further investigated its understanding of exomic regions using eight newly constructed benchmarks based on the ClinVar dataset [33].

#### Evaluation on structural understanding of genomes

**Figure 1:**
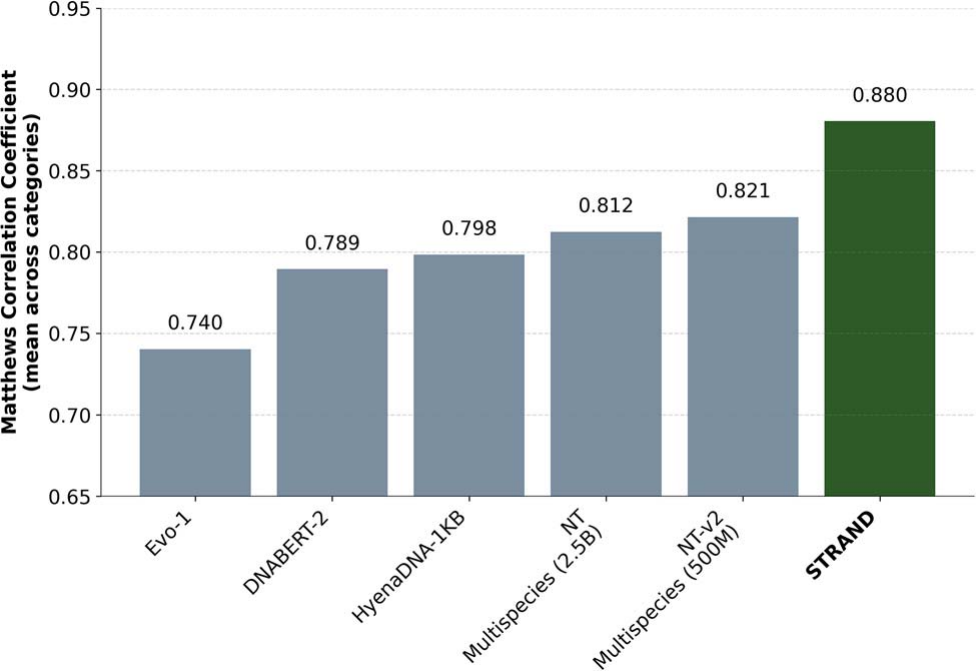
Aggregate MCC score on NT benchmarks evaluated on different genomic models.

We first evaluated STRAND’s fundamental genomic understanding using the established NT benchmarks [14], which comprise 18 diverse molecular phenotype prediction tasks. Our model achieved remarkable improvements over existing state-of-the-art approaches. Our largest STRAND variant (1B parameters) surpassed previous benchmarks, demonstrating an 8.2% improvement over the original NT and a 7% improvement over NT-v2 [14]. STRAND outperformed the second-best model by 0.41% in Splicing, 21.91% in Regulatory, and 26.10% in Chromatin, demonstrating a marginal gain in Splicing but significant improvements in Regulatory and Chromatin predictions (Table 5). All these evaluations were run with 10-fold cross validation, and the median for each evaluation was reported. To further evaluate our STRAND model, we compared its performance with other published models, including HyenaDNA (with 1k sequence length) [15], DNABERT-2 [17, 34, 35], and Evo-1 [15]. Our model significantly outperforms these models, as shown in Fig. 1 and Table 5. (The results in Table 5 are on an older version of the dataset available here: https://huggingface.co/datasets/InstaDeepAI/nucleotide_transformer_downstream_tasks).

**Table 5:** Experimental results on the public NT tasks

Model	NT Paper / Structural		
	Splicing	Regulatory	Chromatin
STRAND	**0.975**	**0.920**	**0.745**
NT-Multispecies, 2.5B	0.973	0.919	0.544
NT-Multispecies v2, 500M	0.974	0.930	0.559
DNABERT-2	0.926	0.910	0.530
HyenaDNA-1KB	0.917	0.880	0.597
Evo-1	0.919	0.705	0.590

Splicing: identification of various splice sites. Regulatory: identification of various regulatory elements. Chromatin: identification of chromatin opening sites. Evaluation using MCC score; best model for each task is shown in bold.

#### Evaluation on variant-level predictions

To evaluate our model’s ability to leverage its understanding of the exomic region to make health-related predictions, we developed new benchmarks using the ClinVar database. In particular, we constructed datasets of nucleotide mutations for seven tasks – five tasks to evaluate exomic enrichment, and the other two being disease-specific tasks. The benchmark evaluations reveal state-of-the-art results in four out of the five broader ClinVar-derived benchmarks (Table 6) and in all RA-specific tasks (Table 7).

**Table 6:** Experimental results on our five ClinVar-derived tasks (MCC)

Model	ClinVar pathogenic					
	CP	HCPS	BRCA	TOP5	PV	Mean
STRAND	**77.4**	**93.7**	**87.7**	99.6	**36**	**78.8**
NT-Multispecies, 2.5B	38.3	71.1	45.3	**99.8**	29.7	56.84
DNABERT-2	58.2	86.8	63.4	99.5	21.1	65.8
HyenaDNA	44.5	81.6	68.5	99.4	13.4	61.5

CP (Cardiovascular Phenotype): identification of variants associated with cardiovascular disease. HCPS (Hereditary Cancer-Predisposing Syndrome): identification of variants associated with cancer predisposition. BRCA: classification of BRCA1, BRCA2, or non-BRCA variants. TOP5 (Top 5 Genes): detection of most-commonly annotated genes. PV (Pathogenic Variant): detection of pathogenic variants. Evaluation of MCC; best model for each task is shown in bold.

**Table 7:** Experimental results on disease specific variant identification tasks (MCC)

Model	Disease-specific variant identification	
	RA variant	IBD variant
STRAND	**0.827**	**0.941**
NT-Multispecies, 2.5B	0.812	0.882
DNABERT-2	0.802	0.926
HyenaDNA	0.794	0.803

RA Variant: identification of variants associated with RA. IBD variant: identification of variants associated with IBD. Evaluation of MCC; best model for each task is shown in bold.

To evaluate the models’ performance in variant-level prediction tasks, we first divided each data set into a training, validation, and test data set. We then attached a classifier head to each model backbone and performed a full fine-tuning on the training set. We used the validation set to perform a hyperparameter search. The values reported in the tables are the models’ test set performance when trained using the optimal hyperparameters.

**Nucleotide mutation benchmark construction:** We derive five new datasets from the ClinVar database [33] that focus on identifying specific mutational effects from nucleotide sequences. These five tasks consisted of: identifying variants belonging to the five most-commonly annotated genes (Top 5 Genes, 43 647 sequences), gene identification given a specific variant (BRCA, 26 438 sequences), disease phenotype prediction from a series of variants (cardiovascular phenotype, 38 224 sequences; hereditary cancer-predisposing syndrome, 70 468 sequences), and predicting clinical significance for a selected number of pathogenic variants (pathogenic variant, 106 843 sequences). The results of our model when fine-tuned on these tasks are reported in Table 6.

**RA-specific benchmark construction:** To simulate the practical and real-world efficacy of our STRAND model for RA and IBD as use cases, we generated two tasks using a similar process. The tasks are as follows: (i) classification of whether a given sequence with a variant is associated with RA (612 RA variants and 38 224 control sequences) and (ii) prediction of whether a given sequence with a variant is associated with IBD (5111 IBD variants and 38 224 controls). These task datasets were used to fine-tune each model using a train, validation, and test distribution of 70%, 12%, and 18%, respectively. The result is reported in Table 7.

**Figure 2:**
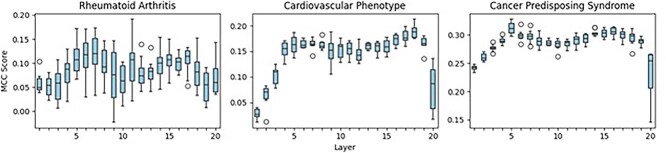
STRAND layer probing experiments performed on binary nucleotide sequence classification tasks. The boxes represent 10-fold cross-validation results for each layer.

**Model robustness.** We evaluate the robustness of our model by performing probing experiments on each intermediate layer. Probing was performed by freezing our STRAND model’s parameters, intercepting the embeddings of the model at each layer, and feeding these embeddings into the input of a simple two-layer multi-layer perceptron classifier. In this way, we trained a unique supervised classifier for each layer. The purpose of probing is to gain insight into the information learned at each layer by comparing the performance of the layers’ classifiers; e.g. a poor predictive model would be overly reliant on certain layers (or have no use for certain layers) for its prediction. We performed these probing experiments on three binary classification tasks: one RA-specific task (prediction of a patient’s RA status, see Evaluation on variant-level predictions), as well as two ClinVar-derived tasks (Cardiovascular Phenotype and Hereditary Cancer-Predisposing Syndrome, see Evaluation on variant-level predictions). We demonstrate the 10-fold cross-validation results in Fig. 2.

Our STRAND model achieves significant advances in genomic sequence understanding, attaining state-of-the-art performance across multiple dimensions of genomic analysis. The model demonstrates exceptional capabilities in both fundamental genomic structure prediction and variant interpretation, with particularly strong performance in disease-specific applications.

## Discussion

This study introduces a novel genetic transformer enriched with exomic data derived from 499 study participants, including patients with and without RA. This is the first study to combine exomes from research participants with rich clinical data in a transformer model, which is also trained with the HRG and other species’ reference genomes. This model has outperformed other available transformers in nearly every task, while maintaining a relatively small size of only 1 billion parameters.

One of the unique features of this model is the use of upstream aligned reads during model training, rather than the more downstream VCF files. This approach mitigates the inductive bias introduced in some of the variant calling and genome-wide association studies processes [36].

The development of this model represents a significant departure from conventional genomic analysis pipelines, and as such, required addressing several key technical challenges. Our findings in model architecture and training strategies have important implications for the field. Most notably, our sequence packing experiments revealed an unexpected advantage of consecutive packing over more complex strategies. This suggests that maintaining the local genomic context is more important for model learning than maximizing position diversity within training windows. This insight could influence future genomic model architectures. Similarly, the transition from BERT-style to GPT-style attention mechanisms mirrors the evolution seen in language models as they scaled, suggesting common principles in large-scale sequence modeling across domains.

Our dynamic loss weighting strategy proved particularly effective in addressing the inherent challenges of genomic data, where the default training approach’s emphasis on frequent patterns is problematic given that rare genetic variants often carry crucial semantic meaning. The success of the strategy in recovering the accuracy lost during data filtration, while improving performance on the HRG, demonstrates the importance of carefully considering the unique characteristics of genomic data in model design. The effectiveness of our data filtration approach in achieving comparable accuracy with significantly reduced computational demands highlights a promising direction for making genomic models more practical for clinical deployment.

The remarkable improvements achieved by our model – an 8.2% improvement over the original NT [14] and a 7% improvement over NT-v2 [14] in fundamental genomic understanding tasks – represent a significant advancement in the field. These gains, particularly the substantial improvements of 21.91% in regulatory and 26.10% in chromatin predictions, suggest that our architectural and training innovations have helped overcome key challenges in genomic modeling. The ability to achieve such improvements while addressing data redundancy and signal-to-noise challenges is particularly significant for clinical applications, where both accuracy and computational efficiency are crucial. These results, combined with the strong performance of the model on disease-specific tasks, suggest that we are approaching a level of genomic understanding that could enable more reliable clinical applications.

Incorporation of multispecies genomes into the training data yielded improvements that suggest a broader applicability across different species and genomic datasets. This, combined with our model’s strong performance across both structural-understanding and variant-level prediction tasks, underscores its potential as a foundation for future applications in disease genomics. The probing experiments further validated the model’s robustness, showing that it learns meaningful representations across its layers, rather than relying heavily on specific layers for predictions.

Our success in both ClinVar-derived tasks and RA/IBD-specific applications is particularly noteworthy. The strong performance across diverse ClinVar tasks – from identifying specific genes to predicting disease phenotypes – demonstrates the model’s ability to capture clinically relevant genetic patterns. The successful application to RA/IBD-specific tasks suggests a promising path for developing disease-specific applications, while the achievement of these results with a relatively small 1B-parameter model indicates potential for even greater improvements with scaled versions. During training the model had not seen any exomes from patients with IBD. Our data scaling experiments, showing consistent improvements as we increased the number of training subjects, further support this potential for growth.

Several strategies exist to improve the performance and clinical applicability of the transformer model. First, expanding the data set by including additional participants from the Tapestry study could enhance the performance of the model, while reducing the risk of overfitting. A more diverse dataset, along with the inclusion of additional exomes representing different clinical cases, would provide valuable insights. The impact of incorporating more data could also help clarify the scaling laws of genomic transformers. Additionally, incorporating human leukocyte antigen typing, as well as accounting for small insertions, deletions, and soft clippings, could further improve the performance and generalizability of the model. These enhancements are currently being developed. Second, refining the model’s ability to make conclusions at the patient level is crucial for clinical application. Efforts are underway to create a workflow that allows for the analysis of individual patient sequences with minimal fine-tuning of classifier layers. For example, a pipeline is being built to identify RA patients from a large cohort using genetic variants associated with autoimmune diseases and RA. Lastly, integrating this model with other types of clinical data, such as clinical notes, imaging, and pathology, is a critical next step in clinical integration. This combination would enable individualized clinical decision making and transform the landscape of medicine. For example, early identification of treatment responders and patients with poor prognoses, such as those at risk for comorbidity, disability, and mortality, is a key priority in the research agendas of prominent rheumatology organizations such as the American College of Rheumatology and the European Alliance of Associations for Rheumatology [26, 37, 38].

There are several potential limitations that should be considered when interpreting the results of this study. As most of the exomes were from white participants from the USA, the results may not be generalized to more racially and ethnically diverse populations. These models will need to be enriched with racial, ethnic, and geographic variation to be more generalizable. Furthermore, these models are considered medical devices under the FDA classification of software as a medical device. Thus, a rigorous clinical validation will be required prior to its implementation in clinical practice.

Our model has the following limitations: The context length is only 1000 bp long, which is on the lower end to allow exons to be fully interpreted without needing multiple inputs. Despite the fact that the model is smaller than most published models, it still takes significant computational power to run and fine-tune. Creating more efficient architectures, securing the models against possible data leaks, and reconstructing the exomes will be a future direction for this research.

## Conclusion

We have developed and validated a first-in-class novel exomic transformer with a preliminary clinical application in RA and IBD. This transformer is able to process and interpret structural and functional genetic variants accurately. This study presents a proof of concept using a novel exomic transformer to process exomic information, expanding the capabilities of the use of artificial intelligence in medicine. The work on testing the performance of this transformer for the identification of patients with RA, IBD, and other diseases across the entire Tapestry study is currently underway and is envisioned to assist in the early and efficient identification of persons with RA and IBD genomic makeups in large biobanks. The applications of this AI tool are broad, and studies on testing the transformer for use in other chronic conditions and for the prediction of the response to anti-rheumatic medications are ongoing. The advent of ever-expanding computational capacities powered by revolutionary algorithms is allowing for the large number of clinical data to be integrated and new models to be created that have the potential to explore the power of genomics and individualize medicine. A careful and ethical approach is required to grow these models in the clinical space and investigate their applicability in diagnosis, prognosis, and therapeutics.

**Table 8:** Characteristics of 499 participants whose exomes were included in the final model^**^

**Characteristic**	**RA**	**Non-RA**	***P*-value**
	**(*N* = 219)**	**(*N* = 280)**	
*Socio-demographics*			
Age, years (mean, SD)	62.21 (13.8)	57.9 (15.7)	.012
Sex, female	171 (78.1%)	187 (66.7%)	.004
***Race/ethnicity***			
Non-Hispanic White	205 (93.6%)	259 (92.5 %)	.256
American Indian or Alaska Native	0	1	
Asian	1	7	
Black or African American	1	5	
Native Hawaiian or Other Pacific Islander	0	0	
Hispanic	8	1	
Other	0	3	
Unknown/Chose not to Disclose	4	4	
***Comorbidities***			
Obesity	90 (41.1%)	40 (14.3%)	.00004
Hypertension	121 (55.2%)	81 (28.9%)	.0041
Diabetes	43 (19.6%)	25 (8.9%)	.344
Hypothyroidism	71 (32.4%)	32 (11.4%)	.0005
Heart disease	65 (29.6%)	62 (22.1%)	.745
Liver Disease	13 (5.9%)	10 (3.5%)	.904
Renal failure	21 (9.5%)	16 (5.7%)	0.771
***RA disease characteristics***			
RA duration (years, mean, SD)	7.95 (5.6)		
RF and/or Anti-CCP positive	128 (58.44%)		
RF positive	94 (42.9%)		
Anti-CCP positive	34 (15.6%)		
ESR at initial visit, mm/hr (mean, SD)	15.2 (15)		
CRP at initial visit, mg/L (mean, SD)	19 (31.5)		
***Medication use***			
Methotrexate use	213 (97.2%)		
Hydroxychloroquine	3 (0.13%)		
TNF inhibitor	35 (15.9%)		
JAK inhibitor	7 (3.1%)		
IL6 inhibitors	8 (3.6%)		
Glucocorticoids use (>5 mg)	148 (67.5%)		

All values are shown as n (%) unless mentioned otherwise and are shown for baseline (defined as RA diagnosis date) ever documented for medications, if documented in Mayo EHR for labs, past medical history. SD: standard deviation, BMI: body mass index, CVD: cardio/cerebrovascular disease, RF: rheumatoid factor, anti-CCP: antibodies to cyclic citrullinated peptide, ESR: erythrocyte sedimentation rate, RA: rheumatoid arthritis, DMARDs: disease modifying anti-rheumatic drugs, erythrocyte sedimentation rate, csDMARDs: conventional synthetic disease modifying anti-rheumatic drugs, bDMARD: biologic DMARD.

## Materials and methods

### Data and computational architecture

The data and computational needs of this project are on the order of several hundreds of gigabytes. We leveraged the Mayo Clinic Cloud (MCC) and the cerebras cluster consisting of up to four CS-3 clusters. Both environments have been reviewed by the organization of PHI compliance.

#### Training data

The model was trained on multiple data sources:

- Human Reference Genome
- Raw sequencing data from Tapestry study participants (1–499 subjects)
- Multi-species genome data

### Exome data: participant cohort

From the Tapestry cohort, a RA subcohort was identified. The Tapestry study is a large, decentralized, clinical Exome assay study of 98 222 adult individuals [39] and is used as the primary source of exomic data for this study. The study excluded patients from the following five states due to state-level regulation: Arizona, Texas, Nevada, Montana, and New York (last known residence). Within the Tapestry study, we have identified a cohort of adults (aged 18 years or older) with RA (*n* = 219) who have at least 2 International Classification of Diseases (ICD) codes for RA + 1 claim for MTX. Two hundred and eighty participants were randomly chosen from the remaining Tapestry cohort (who did not have RA). The details of this cohort are described in Table 8. We extract BAM, FASTQ, and VCF files (used only in the creation of ClinVar and RA datasets for downstream tasks) from the Tapestry study. All relevant clinical data are accessed from the Mayo Clinic electronic health record.

### Data preparation for exomic transformer

Data for transformer training are prepared from BAM and FASTAQ files on MCC. The BAM files will be processed into the BED and FASTQ files. This process is described in detail in Supplementary Methods section and Supplementary Fig. S2. This is followed by filtering and compression to reduce file size without losing information. All alternate contigs are tracked by the contig index and are tokenized. The tokenization process converts the alpha value for each nucleotide into an integer value, enabling proper processing downstream by the exome transformer. To handle each case, we use a different integer value depending on the different contig string matching cases with a dictionary size up to 16.

### Model architecture and training

STRAND is a transformer-based foundation model capable of learning directly from upstream genomic sequences. This approach poses a few technical challenges:

- Information organization: Since 151 base pair reads had to be combined into continuous sequences, multiple different packing strategies were tried. The effectiveness of each of the strategies was evaluated based on the model sequence.
- Data redundancy: To address the significant redundancy inherent to this type of data, we filtered low-quality reads while preserving variant data.
- Signal-to-noise ratio: Dynamic importance reweighting was implemented, inversely weighing each position proportional to their frequency, and assigning higher weights to variant positions.

#### Model architecture

The model is an auto-regressive decoder only transformer trained on next token prediction in a self-supervised model. It consists of 20 layers with a hidden dimension of 1856. We implemented a two-phase training to enhance the model’s learning of the reference genome first, then a mixed training with Tapestry participants.

#### Evaluations and ablations

- Upstream tasks: Accuracy of next token prediction
- Downstream tasks: Consisting of structural genomic understanding (with splice sites, chromatin and regulatory elements [14]) and a set of functional tasks (ClinVar benchmark datasets and RA IBD specific datasets).
- Ablation studies: We evaluated both causal attention mechanisms and masked language modeling attention. However, as the training scale grew in terms of both data and computing budget, language models transitioned to causal attention models that learn via next-token prediction [17, 34, 35].
- Context length: To evaluate the impact of context length on model performance, we conducted experiments using the HRG as our testbed, varying the context length from 600 to 16 384 bp, while maintaining the same number of training tokens. Next-token prediction accuracy showed only modest improvements, increasing from 56.93% at 600 bp to 58.7% at 16 384 bp, and thus a context length of 1024 was chosen.
- Positional encoding: we experimented with four different positional encoding methods; fixed positional encoding [40], relative positional encoding [41], learnable positional encoding [42], and rotary positional encoding [43]. The performance of each method is summarized in Table 9. Rotary positional encoding provided the best performance, demonstrating a favorable balance of accuracy and simplicity.
- Attention window: To investigate the impact of window size, we conducted experiments using window sizes of 256, 512, and 1024 tokens, while maintaining a fixed context length of 4096 tokens. As detailed in Table 10, smaller attention window sizes were associated with a decrease in accuracy.

**Table 9:** Next-token prediction accuracy for different positional encoding methods

**Positional encoding method**	**Accuracy (%)**
Rotary positional encoding	58.75
Learnable positional encoding	56.13
Fixed positional encoding	57.04
Relative positional encoding	58.04

**Table 10:** Next-token prediction accuracy for different attention window sizes

**Attention window size**	**Accuracy (%)**
256	58.57
512	58.61
1024	58.70
4096 (dense)	58.75

#### Benchmarks construction

Specific ClinVar based benchmark datasets were constructed to evaluate the model’s functional understanding. The details are summarized in Supplementary material.

**Key Points**

- STRAND is an exomic-based novel transformer with state-of-the-art performance in various previously published benchmarks while maintaining a relatively small size.
- STRAND is also the first transformer that was applied directly to patient-level data for diagnostic prediction and exceeded other currently published models.
- STRAND’s architecture, data mixture, data packing schemes, and dynamic loss weighting are unique and confer a generalized genomic structural and functional understanding.
- STRAND’s results suggest new possibilities in using relatively upstream data such as binary alignment maps in clinical applications.

## Supplementary Material

Supplement_(October_28)_bbaf618

## Data Availability

Parts of the data could be made available upon review of certain requests. Parts of the code not containing any PHI could be made available for review and reproducibility.
