# Supplementary material for: Introducing a foundational sequence transformer for range adaptive nucleotide decoding (STRAND)
Source: Brief Bioinform. 2025 Nov 24;26(6):bbaf618. doi: 10.1093/bib/bbaf618 (PMC12641612; doi:10.1093/bib/bbaf618)
Supplement: Supplement_(October_28)_bbaf618 [file supplement_(october_28)_bbaf618.pdf]

1 Detailed Methods

1.1 Data and Computational Architecture

The data and computational needs of this project are in the order of multiple hundreds of terabytes. Exomic data are inherently very large, and developing a foundational generative model will require significant computational resources. We leveraged two compute resources available within our institution: The Mayo Clinic Cloud (MCC) and the Cerebras Cluster consisting of up to four CS-3 Clusters. CPU and GPU processes including tokenization where performed leveraging the MCC environment, while training of the model leveraged our CS-3 resources. Both environments have been reviewed by the organization of PHI compliance. The conversion of genomic sequences into tokenized representations enables the analysis of genetic information without access to personally identifiable data, thus preserving the confidentiality of the subject.

1.1.1 Training Data

The model was trained on multiple data sources:

- Human Reference Genome (HRG)
- Raw sequencing data from Tapestry study participants (1-100 subjects)
- Multi-species genome data

For multispecies training, we experimented with different mixing ratios:

- 50:50 ratio with 15% of multi-species data (half of the entire input data was from HRG and the other half was from multi-species using a total of 15% of the multi-species data).
- 14:86 ratio with 100% of multi-species data (with 14 parts being from HRG and 86 parts from multi species using all of the available multi-species data).

Training experiments evaluated the impact of both data quantity (number of subjects) and diversity (inclusion of multi-species genomes) impacts on model performance. To assess scalability, we systematically increased the number of subjects in the training data from 1 to 100, evaluating performance on both HRG and Tapestry datasets at each step. Once a final architecture and training recipe was chosen, a total of 499 participant exomes were added to the training dataset. The incorporation of multi-species genomes was evaluated to assess the model's ability to learn from broader genomic contexts and its potential for generalization across different species.

1.2 Exome Data: Participant Cohort

From The Tapestry cohort a RA subcohort was identified and is described below.

1.2.1 Tapestry Cohort

The Tapestry study is a large, decentralized, clinical Exome assay study of 98,222 adult individuals[1] and is used as the primary source of exomic data for this study. All available exomes, stored on the omics data platform (ODP), as part of MCC. All patients enrolled in Tapestry with active consent who have their exome available in the ODP were included in the study. The study excluded patients from the following five states due to state-level regulation: Arizona, Texas, Nevada, Montana, and New York (last known residence). This is the basis of the training/testing data used in the development of the models. Of the available exomes a total of 499 patients were chosen. First, all patients with RA (subcohort below) were excluded from the main Tapestry cohort and a cohort of them (219 total) were randomly selected and included in the subcohort. The participants in the rest of the Tapestry study were then randomly selected to round the number of participants to 500. One participant had to be removed as their BAM files were not readily accessible.

1.2.2 Rheumatoid Arthritis subcohort

Within the Tapestry study, we have identified a cohort of adults (aged 18 years or older) with RA (n = 619) who have at least 2 ICD codes for RA + 1 claim for MTX. This subcohort was manually reviewed for confirmation of their RA diagnosis by a group of trained registered nurses. All questionable cases were additionally evaluated by two physicians (SA and EM). The characteristics of the patient and the rheumatologic history were extracted from the electronic health record (EHR). This cohort was used as the basis for validation of the model's performance. The details of this cohort are described in Table 8 in main document.

1.3 Data Access for Exomic Transformer Design and Training

We used the data of the Tapestry study to access full raw exomes on all participants. We extract BAM, FASTQ and VCF files (used only in the creation of ClinVar and RA datasets for downstream tasks) from the Tapestry study ODP repository for included participants. The Tapestry data were transferred through a secure Google Cloud link to the storage buckets of this project. In addition, the human reference genome (GRCh38.p14, Human Genome Overview - Genome Reference Consortium) and whole genomes of various species available through the NIH on an open access basis were downloaded to enrich the training set and add multispecies genomes. (Genome - NCBI - NLM)

1.4 Data Preparation for Exomic transformer

Data for transformer training is prepared from BAM and FASTAQ files on MCC.

1.4.1 BAM to BED and FASTQ

Using a well-established bioinformatics pipeline, the BAM files will be processed into the BED and FASTQ files [2]. This process is described in detail below.

BAM files are binary files that store aligned reads from sequencing platforms and contain information such as read name, sequence, quality, and alignment position. BED files are text files that store genomic regions of interest, such as genes, exons, or variants, and contain information such as chromosomes, start, end, name, score, and strand. The transformation process from BAM to BED involves the steps described below.

Bedtools bamToBed was used to convert BAM files to BED files and specify parameters such as minimum mapping quality, CIGAR score, and read length standardized to 151 bp. Using a custom BED2BED tool to sort, parse and filter the BED files, and specify the parameters such as minimum region length, maximum region length, and minimum region score. This is an in-house developed bed processing Python script to filter to cigar-151M reads and transform reads to the positive strand. Additionally, tagging and parsing of bed entries containing insertions and deletions or minor soft-clipping. Insertions and deletions are filtered to a single event that is 20bp or less for confidence of being captured within a single read, as well as a maximum of 20bp soft-clipped from the 5 ' or 3' end of the read.

Picard SamToFastq tool is used to convert the BAM files to FASTQ files and specify parameters such as the reference genome. MD5SUM checks were performed as part of all cloud-based transfers. The created files are stored in a separate bucket in the Mayo Cloud and labeled.

1.4.2 Filtration and Compression

The next step involves filtering and compression to reduce file size without losing information. The filtering and compression algorithm for the BED and FASTQ files occurs at the individual level of the BED and FASTQ file of the subject. After processing of the BAM files, two 2 BED files and two FASTQ files are produced that are used in the filtering and compression process.

The following rules are applied to each BED row we process to retrieve, trim, and align each genomic sequence:

- Confirm Final Cigar Score Has a M (matching score) of greater than or equal to 131
- Use the Read Name string from the BED file row to lookup the 151-character (A, C, T, G, N values) sequence from the FASTQ file.
- Apply the substring trimming method to the 151-character long sequence.
- If soft clip/insertion/deletion, pad to 170. Filtering reads to only ONE cigar change

- Check the strand score and apply the reverse complement if the strand is denoted as a negative (-) strand to properly align them.

- All alternate contigs are tracked by the contig index and are tokenized. Contig index added to the bed file so a separate token for the contig index and name is generated.

1.4.3 Tokenization Process

The tokenization process converts the alpha value for each nucleotide into an integer value enabling proper processing downstream by the exome transformer. To handle each case, we use a different integer value depending on the different contig string matching cases with a dictionary size up to 16. Insertions and deletions were not considered and these BAMs were filtered out.

151 Match Case

All 151M sequences are organized in their own BED file. In this case, we only need to confirm that the cigar score is a 151M match and then use the read name to find the proper sequence from the FASTQ file and then generate the tokenization. Once the sequence is found, we then check the strand value, which can be - or +, enabling us to know when to do the reverse complement of the sequence enabling proper alignment across all sequences before finally tokenizing each individual nucleotide. The start location of every sequence is tracked from the BED file with a separate token for the start position.

1.4.4 Data Transfer to Cerebras Cluster

Once the tokenized NPZ files are generated, they are securely transferred to the Mayo Cluster on the Cerebras side. These files are anonymized, and the unique identifier is maintained only on MCC.

1.4.5 Clinical Data Access through Mayo EHR

For the rheumatoid arthritis cohort identified by the enterprise-wide search of the EHR, patient information is securely maintained on MCC through multiple BigQuery tables (Google Cloud. (n.d.). The only patient identifier is the medical record number. Other identifiers are removed from the data. The data includes laboratory tests relevant for RA (biomarkers, CBC data, BMP data, cultures, etc.), basic demographics, medical history and problem list entries, medications and medication orders, encounters and encounter dates.

1.5 Model Architecture and Training

1.5.1 Model Overview

is a transformer-based foundation model designed to process and understand aligned upstream genomic sequencing data directly, without requiring traditional preprocessing steps [3]. The development of a model capable of learning directly from upstream genomic sequences represents a significant departure from conventional

genomic analysis pipelines and presented several technical challenges that influenced our architectural decisions and training methodology. These challenges are particularly relevant for clinical genomics applications where rapid and accurate variant detection is crucial. We describe these challenges and our solutions below, followed by comprehensive performance analyses.

1.5.2 Core Technical Challenges

Information Organization

Raw genome sequencing data comes in short 151-base-pair reads that cannot be trivially combined into continuous sequences. This required specialized data organization strategies to maintain long-range dependencies. To process these reads, we developed several packing strategies to organize the input data into fixed-size context windows:

- **Consecutive Packing:** Reads from the same chromosome were packed in ascending order of genomic position.
- **Random Packing:** Reads from the same chromosome were randomly packed into contexts.
- **Overlapping Packing:** Adjacent reads were merged into the longest possible contiguous sequences.
- **Spaced Packing:** Adjacent non-overlapping reads were packed to ensure uniform distribution of genomic positions.

The effectiveness of each packing strategy was evaluated based on the model's accuracy in predicting the next base pair in a sequence. We performed comprehensive all-to-all comparisons by training models on data prepared with each packing strategy and evaluating them on datasets prepared with each strategy, as well as the HRG dataset. Our initial hypothesis was that Overlapping or Spaced packing schemes would perform better due to their incorporation of domain-specific knowledge and ability to pack more unique information within a context.

Data Redundancy

Raw sequencing reads contain significant redundancy, with coverage depth varying from 1 to 180 reads per genomic position. Within each 151-base-pair read, typically only 1-2 base pairs differ from the reference genome. To address this redundancy, we implemented a filtration strategy to remove low-quality reads while preserving variant information. All of the HRG reads were included in the final dataset. However, only reads containing at least one variant were included from the rest of the patient level input data. This approach achieved an 8-fold reduction in training data volume while maintaining model performance.

Signal-to-Noise Ratio

Genetic variants represent a small fraction of total base pairs in the data. This creates an imbalance where the model could achieve high accuracy by memorizing reference sequences while ignoring rare variants. To address this challenge, we implemented a dynamic importance re-weighting strategy based on two factors:

- **Reference Bias Correction:** Base pairs were weighted inversely proportional to their frequency at each position in the training data.
- **Variant Emphasis:** Higher weights were assigned to variant positions to enhance their contribution during training.

This weighting strategy was mathematically implemented as:

$$\text{loss}_{\text{context}} = \sum_{i=0}^{\text{context\_length}} \text{loss}(i) \times \text{coverage\_weight}(i) \times \text{match}_{\text{HRG}}(i)$$

where:

- $\text{loss}(i)$: Cross-entropy error at context position i
- $\text{coverage\_weight}(i)$: Inverse of coverage depth $1/\text{depth}_{\text{chr,pos}}(i)$ for the genomic position
- $\text{match}_{\text{HRG}}(i)$: α for reference matches, β for variants (where $\beta > \alpha$)

1.5.3 Model Architecture

This model uses an autoregressive, decoder-only transformer architecture trained on next-token prediction in a self-supervised manner. The architecture consists of 20 layers with a hidden dimension of 1856 and employs 16 attention heads using Multi-Query Attention [4] with 8 KV groups. The feed-forward network uses SwiGLU activation with a dimension of 7424. For positional information, we implement Rotary Position Encoding (RoPE) with 116 rotary dimensions. The model processes sequences with a context length of 1024 tokens and uses a compact vocabulary of 16 tokens. Layer normalization is handled through RMSNorm ($\epsilon = 1e^{-5}$), and the model comprises approximately 1 billion parameters in total. Training leverages conditional BFloat16 mixed precision for computational efficiency.

For optimization, we employed the AdamW optimizer ($\beta_1 = 0.9$, $\beta_2 = 0.95$, $\epsilon = 1e^{-8}$) with a weight decay of 0.1.

1.5.4 Training Protocol

The model was trained in two distinct phases:

- **Phase 1:** Initial training on HRG and multi-species genomes using both upstream and downstream evaluations. Training used a global batch size of 512 sequences with a mixture of human reference genome (13.34%) and multi-species genomes (86.67% from 786 species). The learning rate followed a two-phase schedule: linear warmup to $4.8e^{-4}$ over 1,000 steps, followed by cosine decay to $4.8e^{-5}$ over 290,000 steps.
- **Phase 2:** Mixed training with Tapestry subject data, HRG, and multi-species genomes across two epochs. Training used a global batch size of 4,096 sequences. Data mixture consisted of Tapestry subject data (75%), multi-species genomes (21.67%), and reference genomes (3.33%). The learning rate warmed up to $1.2e^{-4}$ over 1,430 steps, followed by cosine decay to $1.2e^{-6}$ over 284,570 steps.

This two-phase approach was chosen because the percentage of information unique to each subject with respect to the human reference genome is extremely small (<<1%). By first training on reference genomes, the model develops a strong understanding of reference genomes before focusing on the rare but important variations between subjects.

1.5.5 Evaluation Metrics

Model performance was assessed using:

- **Upstream Tasks:** Accuracy of predicting the next nucleotide in a sequence
- **Downstream Tasks:** Recognition of structural elements (including chromatin and regulatory elements) and benchmarking against ClinVar variants

The downstream tasks span three main categories of structural understanding:

- **Splicing:** Identification of various splice sites
- **Regulatory:** Identification of various regulatory elements
- **Chromatin:** Identification of chromatin opening sites

Additionally, we evaluated the model on variant-level predictions using ClinVar-derived benchmarks and RA-specific tasks to assess its clinical applicability.

1.5.6 Ablation Studies

We conducted extensive ablation studies to determine optimal model architecture and training configurations. Key architectural components were systematically evaluated:

Attention Mechanism

We evaluated both causal attention and masked language modeling attention mechanisms. The causal attention approach uses next-token prediction, where the model can only attend to previous positions in the sequence, while the masked language modeling approach used 15% random masking of input tokens, allowing bidirectional attention across the entire sequence. This design choice was influenced by the increasing scale of available genomic training data and the success of similar approaches in language models. In the masked language modeling approach, the model is trained to "fill-in-the-blanks" by predicting randomly masked tokens, mirroring early self-supervised language models [5–7]. However, as training scale grew in terms of both data and compute budget, language models transitioned to causal attention models that learn via next-token prediction (asking the model to "complete the sequence"). Given the significant increase in genomic data available for training, we conducted comparative experiments using both approaches to determine the most effective mechanism for genomic sequences.

Context Length

Context length refers to the number of tokens a model can process at once. is trained on a combination of the human reference genome (HRG), 786 multi-species genomes, and the raw sequencing data from 500 Tapestry participants. While the raw sequencing data is fragmented into 151-base-pair reads, the primary benefit of increased context length comes from processing contiguous sequences in reference genomes rather than the fragmented sequencing data. To evaluate the impact of context length on model performance, we conducted experiments using the human reference genome as our testbed, varying the context length from 600bp to 16384bp while maintaining the same number of training tokens. Next-token prediction accuracy, which measures the model's ability to correctly predict the next base in a sequence, showed only modest improvements, increasing from 56.93% at 600bp to 58.7% at 16384bp. Given these results, we chose a context length of 1024 tokens as an optimal balance between computational efficiency and modeling capability.

Positional Encoding

Positional encoding in transformer models provides information about the relative position of tokens within the sequence. These positions refer to the token's location in the sequence rather than the actual physical positions of the base pairs. For , we experimented with four different positional encoding methods: fixed positional encoding[8], relative positional encoding[9], learnable positional encoding[10], and Rotary Positional Encoding[11]. The performance of each method is summarized in main document. Rotary Positional Encoding provided the best performance, demonstrating a favorable balance of accuracy and simplicity.

Attention Window Configuration

To manage the computational demands of processing long genomic sequences, we explored a sliding window attention mechanism[12]. This mechanism restricts the attention computation to a fixed-size window of tokens, which slides across the input sequence, thereby enhancing computational efficiency and reducing memory requirements. We hypothesized that beyond these practical benefits, a sliding window approach might encourage the model to learn hierarchical representations of genomic sequences, potentially improving its ability to capture long-range dependencies relevant to genomic function. To investigate the impact of window size, we conducted experiments using window sizes of 256, 512, and 1024 tokens, while maintaining a fixed context length of 4096 tokens. As detailed in main document, our results indicate that employing smaller attention windows led to a decrease in next-token prediction accuracy, without substantial gains in computational efficiency within the tested range.

Additional Parameters

We also systematically evaluated:

- Masking levels (0-30%)
- Data filtration thresholds (coverage depth ¡10x, HRG mismatch ratio ¡10%)

1.6 Benchmarks Construction

In order to capture the unique characteristics of the exome, correct genetic variant detection and interpretation was tested by constructing specific ClinVar data sets. Each dataset was prepared by identifying the exomic locations of task-specific variants through ClinVar metadata, overlaying them on the HRG, and extracting nearby nucleotides within a 100-wide context window to generate sequences. Finally, we filtered out all duplicate rows. In total, 6.13 million variants were loaded from ClinVar and filtered for HRG38 and SNVs, leaving 2.77 million variants. Then, we filtered for only germline variants from valid chromosomes.

Next, these variants were combined with GENCODE data [13] to create a BED format dataset, effectively resulting in a unique exomic ClinVar dataset of 2.351 million variants and associated metadata (e.g., genome location, phenotype, and gene symbol). We used this dataset to derive each of the initial five tasks for evaluation of the models' performance on pathogenicity and disease states, described in results section above.

The second portion consisted of a more focused evaluation of RA and IBD with the creation of two different tasks. First, we identified the exomic locations of variants associated with RA from ClinVar and overlaid them on the HRG at each exomic location, and extracted nearby nucleotides within a 200-wide context window. The variants were isolated according to the two tasks by cross-referencing the ClinVar variant metadata. Finally, all duplicate rows were filtered out. The same task was repeated for the IBD variants. Evo1 was not included in the testing of the variant tasks given observed persistently high gradient norms when training with Evo1, which consistently results in an MCC score of 0. Despite experimenting with several mitigation strategies—including reducing the learning rate (to 1e-4 and 1e-6), applying gradient clipping, adjusting weight initialization, and other standard techniques—the issue remains. All classifiers were trained on a split of the data to train, validation, and test distribution of 70%, 12%, and 18%, respectively. Ten fold cross validation was performed to ensure robustness and reproducibility.

References

[1] Bandel, L. A. *et al.* Mayo clinic tapestry study: A large-scale decentralized whole exome sequencing study for clinical practice, research discovery, and genomic education. *Mayo Clin Proc* (2024).

[2] Quinlan, A. R. & Hall, I. M. Bedtools: a flexible suite of utilities for comparing genomic features. *Bioinformatics* **26**, 841–842 (2010). URL https://doi.org/10.1093/bioinformatics/btq033.

[3] Zou, J. *et al.* A primer on deep learning in genomics. *Nat Genet* **51**, 12–18 (2019).

[4] Ainslie, J. *et al.* Gqa: Training generalized multi-query transformer models from multi-head checkpoints (2023). URL https://arxiv.org/abs/2305.13245. 2305.13245.

[5] Ji, Y., Zhou, Z., Liu, H. & Davuluri, R. V. Dnabert: pre-trained bidirectional encoder representations from transformers model for dna-language in genome. *Bioinformatics* **37**, 2112–2120 (2021).

[6] Zhou, Z. *et al.* Dnabert-2: Efficient foundation model and benchmark for multi-species genome. *ArXiv* **abs/2306.15006** (2023).

[7] Zhou, Z. *et al.* Dnabert-s: Pioneering species differentiation with species-aware dna embeddings. *ArXiv* (2024).

[8] Zhu, J. *et al.* Rethinking addressing in language models via contexualized equivariant positional encoding (2025). URL https://arxiv.org/abs/2501.00712. 2501.00712.

[9] Qin, Z. *et al.* Linearized relative positional encoding (2023). URL https://arxiv.org/abs/2307.09270. 2307.09270.

[10] Li, Y., Si, S., Li, G., Hsieh, C.-J. & Bengio, S. Learnable fourier features for multi-dimensional spatial positional encoding (2021). URL https://arxiv.org/abs/2106.02795. 2106.02795.

[11] Su, J. *et al.* Roformer: Enhanced transformer with rotary position embedding (2023). URL https://arxiv.org/abs/2104.09864. 2104.09864.

[12] Li, Y., Chen, J., Ma, J., Wang, X. & Zhang, W. Gaze estimation based on convolutional structure and sliding window-based attention mechanism. *Sensors* **23**, 6226 (2023). URL https://www.mdpi.com/1424-8220/23/13/6226.

[13] Harrow, J. *et al.* Gencode: the reference human genome annotation for the encode project. *Genome Res* **22**, 1760–74.

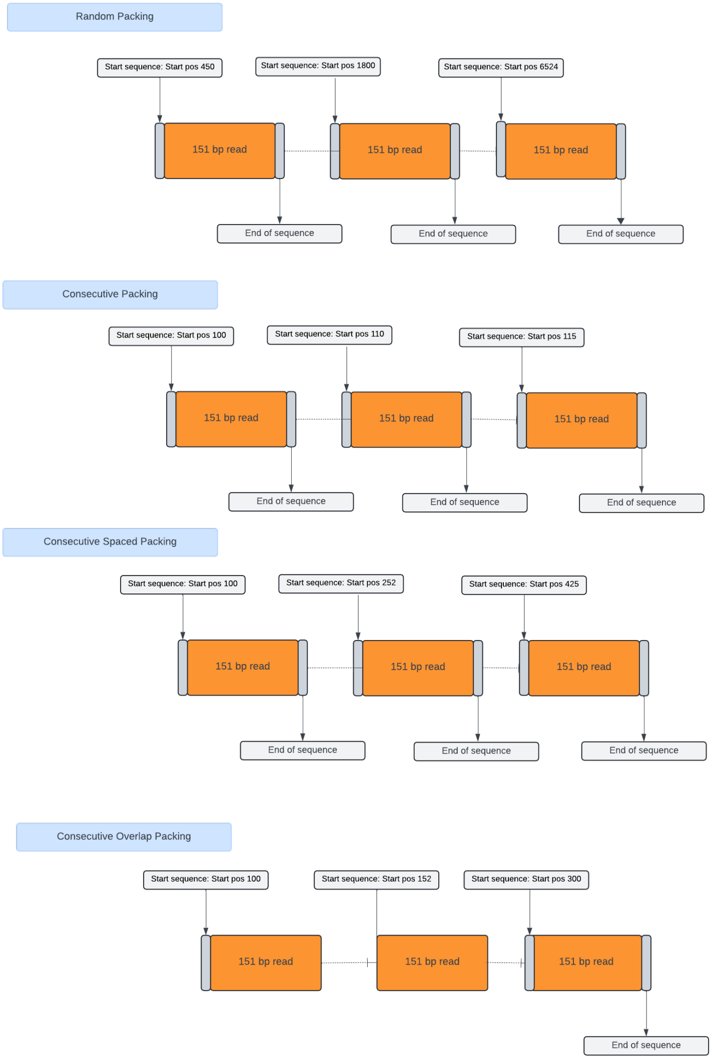

Fig. 1 Visual Display of different packing strategies

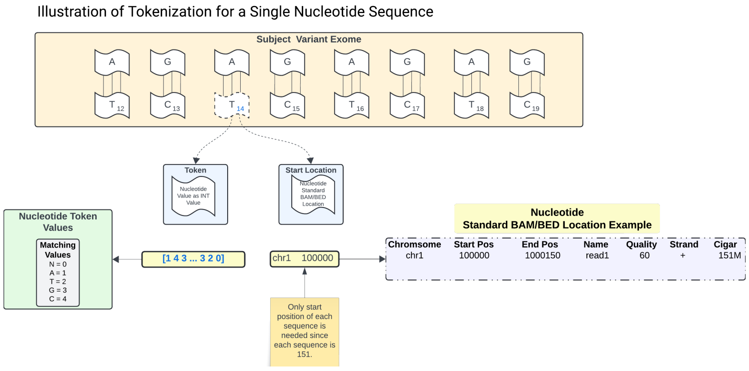

Fig. 2 Tokenization approach
